# Supplementary material for: Optimizing Precision Probiotics for Mitigating Graft-Versus-Host Disease
Source: Microorganisms. 2025 Mar 21;13(4):706. doi: 10.3390/microorganisms13040706 (PMC12029423; doi:10.3390/microorganisms13040706)
Supplement: Supplementary file 1 [file microorganisms-13-00706-s001.zip › Figure S1.pdf]

Figure S1. 16s rRNA sequencing identifies specific bacterial differences in mice after stem cell transplant. (**A**) Taxonomic composition of bacteria taxa over time from select fecal specimens of mice over time, pre and post stem cell transplant, and following treatment. Each vertical bar represents 1 individual mouse. (**B**) Linear discriminant analysis effect size measurements (LEfSe) of bacterial taxa effect enriched in groups that received the standard probiotic blend versus the optimized probiotic blend.
