# Supplementary material for: Optimizing Precision Probiotics for Mitigating Graft-Versus-Host Disease
Source: Microorganisms. 2025 Mar 21;13(4):706. doi: 10.3390/microorganisms13040706 (PMC12029423; doi:10.3390/microorganisms13040706)
Supplement: Supplementary file 1 [file microorganisms-13-00706-s001.zip › Figure S2.pdf]

Figure S2. Bacterial group levels in mice with graft-versus host disease (GVHD) treated with vehicle (grey), standard probiotics (blue), and optimized probiotics (maroon). Bacterial group qPCR (log10 copies/g feces) performed on gDNA isolated from fecal specimens collected from mice that underwent allogeneic stem cell transplantation and developed GVHD. (**A**) CLEPT (*Clostridium leptum* group, Phylum Firmicutes, Clostridial Phylogenetic Cluster IV) and (**B**) ENTERO (Phylum Proteobacteria, Family Enterobacteriaceae) levels at timepoints throughout the course of the experiment measured. Each qPCR assay was performed in triplicate. Bars represent the mean ± SEM. Points represent results from individual animals. Statistical analysis by Mann-Whitney test. * $P < .05$. ** $P < .01$. ***$P < .001$. NS, not significant.
