# Supplementary figures and images for: Optimizing Precision Probiotics for Mitigating Graft-Versus-Host Disease
Source: Microorganisms. 2025 Mar 21;13(4):706. doi: 10.3390/microorganisms13040706 (PMC12029423; doi:10.3390/microorganisms13040706)

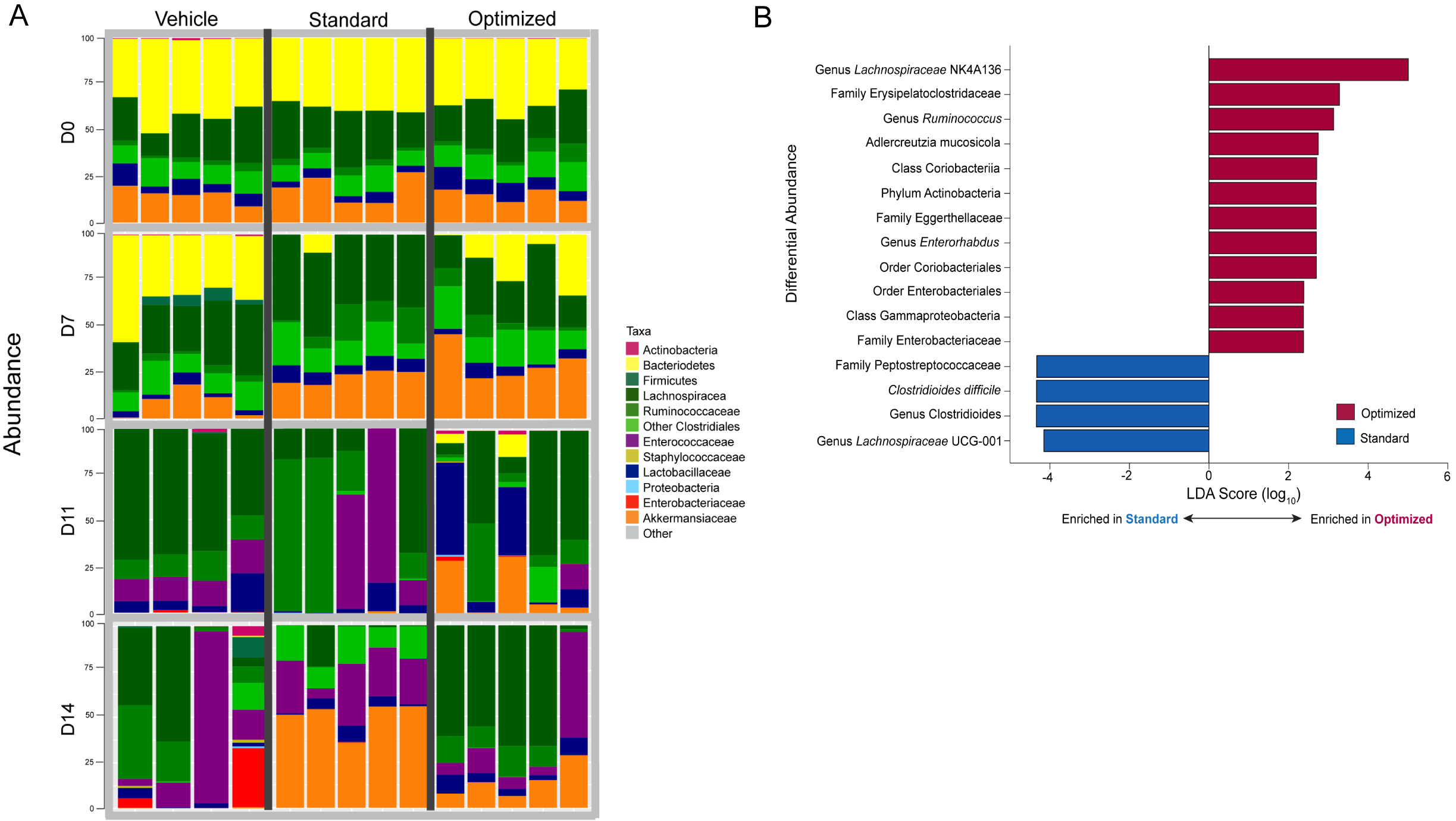

Supplement: Supplementary file 1 [file microorganisms-13-00706-s001.zip › Figure S1.tif]

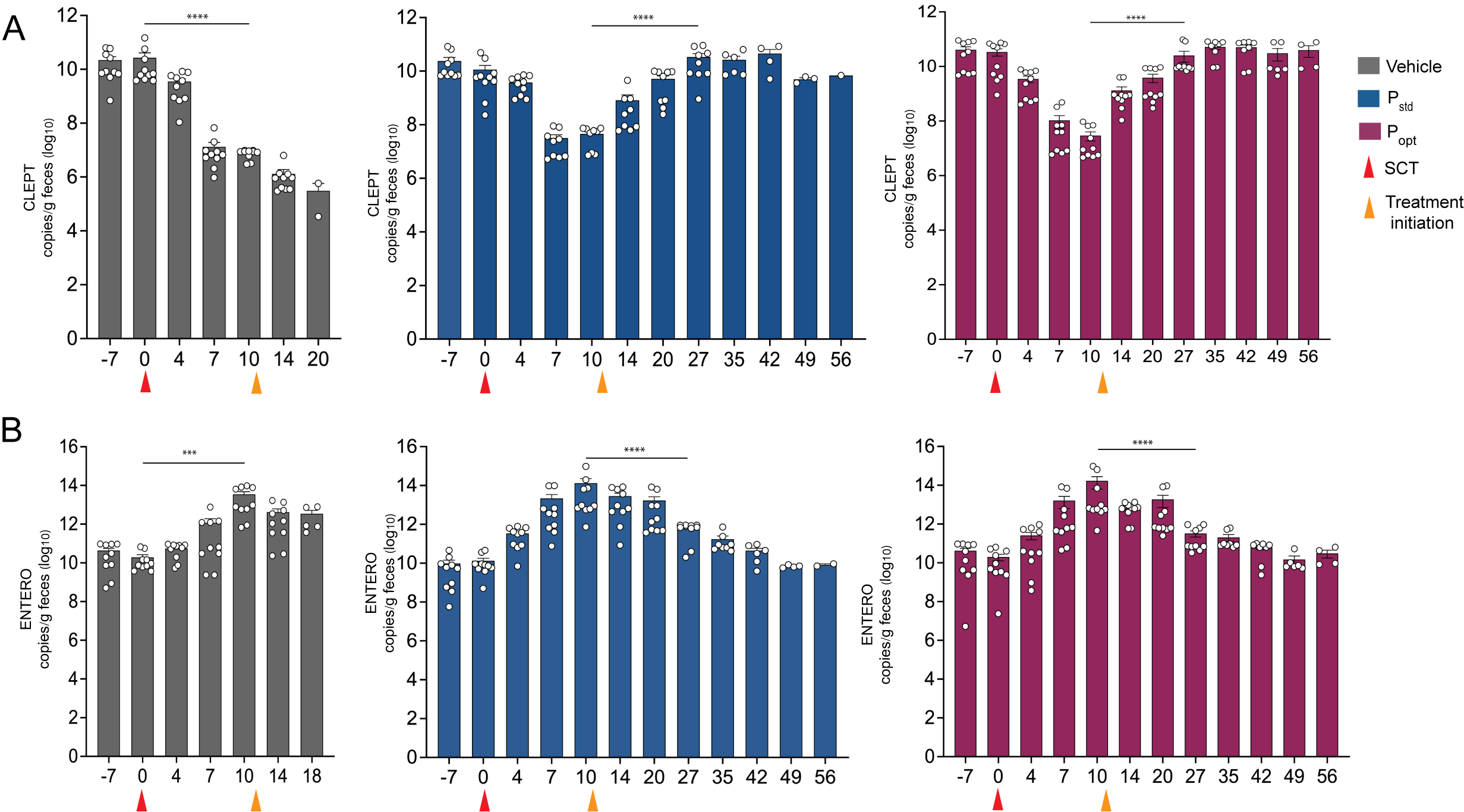

Supplement: Supplementary file 1 [file microorganisms-13-00706-s001.zip › Figure S2.tif]
